# Retrospective Comparison of Complication Rates Following Tibiotalocalcaneal (TTC) Nail Fusion vs ORIF for Neuropathic Ankle Fractures

**DOI:** 10.1177/24730114261425629

**Published:** 2026-03-10

**Authors:** Spencer C. DeMedal, Kelly Dopke, Kelan Queenan, Samantha N. Olson, Michael F. Levidy, Tonya S. King, Jana Davis, Michael C. Aynardi

**Affiliations:** 1 The Pennsylvania State College of Medicine, USA; 2 University of California San Diego, USA; 3 Penn State Health Milton S. Hershey Medical Center, USA; 4 Sarasota Memorial Healthcare System, FL, USA

**Keywords:** Neuropathic fracture, Tibiotalocalcaneal arthrodesis, Hindfoot fixation

## Abstract

**Background:**

Ankle fractures occurring in the setting of underlying neuropathy pose a significant risk for complications. Poor clinical outcomes such as primary failure of an operative surgical construct, wound infection, ulceration, and osteomyelitis are increased in neuropathic diabetic patients. Operative management includes open reduction internal fixation (ORIF) vs a fusion of the ankle using a tibiotalocalcaneal (TTC) nail. This study aims to compare clinical outcomes and complication profiles following operative fixation of neuropathic ankle fractures between patients undergoing ORIF and those undergoing TTC fusion.

**Methods:**

Institutional review board approval was obtained to create a retrospective database of patients over a 17-year period. Included patients had a diagnosis of neuropathy of the lower extremity and an ankle fracture requiring either ORIF or TTC fusion. Demographic and clinical data were collected for the study sample. Descriptive statistics were conducted, and Kruskal-Wallis and χ^2^ tests were used for analysis.

**Results:**

Forty-five patients were included in the study, of which 26 underwent ORIF and 19 had a TTC fusion. There was a significant difference in weightbearing status at 2 weeks postoperatively between the 2 groups (*P* < 0.01), with 80.8% of ORIF patients and 36.8% of TTC fusion patients being nonweightbearing (NWB). However, there was no significant difference in the weightbearing status between ORIF and TTC fusion patients (*P* > .05) at later time points. Postoperative complications (*P* > .99) and use of opioid medications (*P* > .99) were not statistically significant when comparing ORIF to TTC fusion with no clear difference detected.

**Conclusion:**

In patients with neuropathic fractures, TTC fusions were associated with earlier enhanced weightbearing capabilities compared to ORIF. This suggests that electing for a TTC fusion instead of an ORIF may provide the patient with improved function earlier in their recovery process, but we detected no clear difference in long-term postoperative outcomes when comparing TTC fusion and ORIF procedures.

**Level of Evidence:**

Level III, retrospective cohort study.

## Graphical abstract

**Graphical Abstract:**
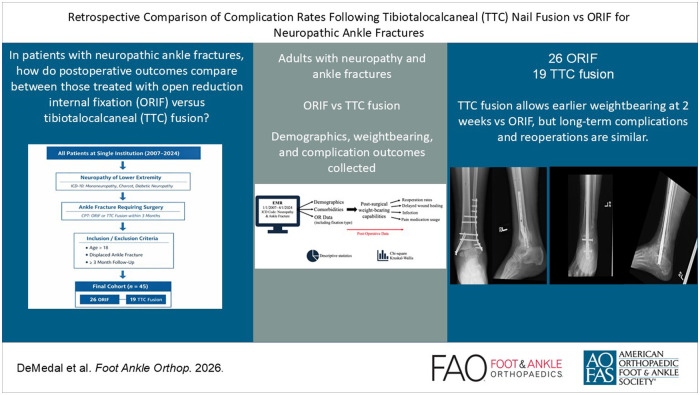

## Introduction

Neuropathic ankle fractures are painless and progressively destructive conditions that have increased prevalence among diabetic populations.^1^ Prolonged microtrauma to skin, soft tissue, and joint space can lead to ulceration, infection, instability, amputation, and even death from these complications.^2,3^ Management of neuropathic fractures includes operative and nonoperative options although both may be associated with their own respective complications.^4^ In particular, operative management of neuropathic ankle fractures in diabetic patients pose significant risks for increased complications such as infection, malunion, nonunion, and amputation when compared to nondiabetic patients.^4,5^ Despite the body of literature that supports this, there are few evidence-based guidelines for operative management of neuropathic fractures.^6^ The decision-making process regarding management is thus shared between the clinician and the patient where the fracture is assessed in combination with the clinical profile of the patient for which a plan of treatment is then determined.^7^

The risk of postoperative complications after operative intervention of any kind remains high, but it remains unclear if open reduction internal fixation (ORIF) or tibiotalocalcaneal (TTC) nail fusion provides better patient outcomes in those with neuropathic ankle fractures. ORIF has long been used for neuropathic fractures, but it is generally associated with more postoperative complications because of wound healing complications and prolonged periods of nonweightbearing following surgery.^8^ Operative planning for a patient with diabetes or neuropathy of the lower extremity often uses double-fixation methods to increase the rigidity of the construct and increases the time to weightbearing to provide increased stability. Alternatively, TTC fusion is a viable salvage procedure for complex diseases involving both the tibiotalar and subtalar joints, including Charcot neuroarthropathy.^9^ Numerous biomechanical studies^10-13^ have demonstrated the rotational stability and strength of intramedullary nails, which may contribute to their utility in the management of complex joint deformities.^14^ Although TTC fusions may improve patient mobility in the early postoperative period, they may have increased risks of complications in diabetic patients, especially those with Charcot neuroarthropathy, poor glycemic control (HbA_1c_ > 7.5), or those with ASA classification >2.^15^ As such, this study seeks to compare clinical outcomes and the complication profile following operative fixation of a neuropathic fracture for those undergoing ORIF vs TTC fusion.

## Methods

Following institutional review board approval for experimental design and protocols, a database of all patients from January 1, 2007, to April 1, 2024, at a single academic medical center with *Current Procedural Terminology* (*CPT*) and *International Classification of Diseases* (*ICD*) codes was created. Prior to database creation, the research network, TriNetX, was used to preliminarily identify patients at a single institution who met inclusion criteria as described below. *ICD-10* and *CPT* codes for mononeuropathy, Charcot neuroarthropathy, or diabetic neuropathy were used as a way to identify patients aged >18 years with a diagnosis of neuropathy of the lower extremity. Of these patients, *CPT* codes for arthrodesis or open treatment within 3 months of fracture were used to preliminarily identify patients. Any patient that met criteria for these parameters was included in the study. Once the patients were identified, the included fracture types for the study were displaced fractures of lateral malleolus or medial malleolus and also included bimalleolar, trimalleolar, and distal fibular fractures.

Once the final database was created from the patients identified on TriNetX using the above parameters, it was further characterized to capture demographic and clinical variables of these patients. The demographic and clinical data that were collected for the study sample included age, sex, race, insurance, county of residence, body mass index (BMI), and patient comorbidities. Patient comorbidities were further categorized using a systems-based approach including vascular (defined as hypertension, congestive heart failure, coronary artery disease, peripheral vascular disease, and/or hyperlipidemia), rheumatologic, neurologic, pulmonary, renal, and endocrine with a delineation for diabetes and a diagnosis of Charcot neuropathic arthropathy. In addition, tobacco use was also recorded. Clinical information included both procedural and postoperative complication data. Procedural variables included date of surgery, fixation type (ORIF, TTC nail, or TTC bail with additional fixation), indication if there was joint preparation, tourniquet and procedure time, blood loss, and number of plates and/or screws used in the final construct.

Postoperative data recorded included time to discharge in days, postoperative narcotic prescription differentiating between opioid vs nonopioid medication, and weightbearing status at different time periods in the postoperative period (0-2 weeks, 4-6 weeks, and at 12 weeks postoperatively). Weightbearing status was categorized into 4 groups: nonweightbearing (NWB), toe-touch weightbearing (TTWB), weightbearing as tolerated (WBAT), and weightbearing (WB). WBAT and WB designations were for patients who were free to bear weight during specific time periods postoperatively. Postoperative weightbearing status was determined by the treating surgeon based on clinical judgment and perceived stability of the construct. Weightbearing status was extracted from the electronic medical record and recorded according to documented postoperative instructions rather than observed patient behavior. A minimum follow-up period of 3 months was established, and complication data included the presence of the following at any time point for the included dates of the study: nonunion, failed hardware, infection, requirement of a reoperation, and readmission to the hospital. Finally, reoperations were further classified by time from original surgery date or from the date of last surgery if they required multiple procedures and whether the following operative variables were performed: removal of hardware, irrigation and debridement, application of a wound vacuum, placement of an external fixator, use of an antibiotic spacer or intramedullary rod, amputation, or conversion to a fusion.

### Statistical Methods

Descriptive statistics were used to summarize the demographic and clinical characteristics of the study sample. Categorical measures were compared between the ORIF and TTC fusion cohorts using Fisher exact tests, and continuous measures were compared between the cohorts using Kruskal-Wallis tests. Significance was defined as *P* < .05. Statistical analyses were performed using SAS statistical software version 9.4 (SAS Institute Inc., Cary NC).

## Results

### Patient demographic data

**Figure 1.**
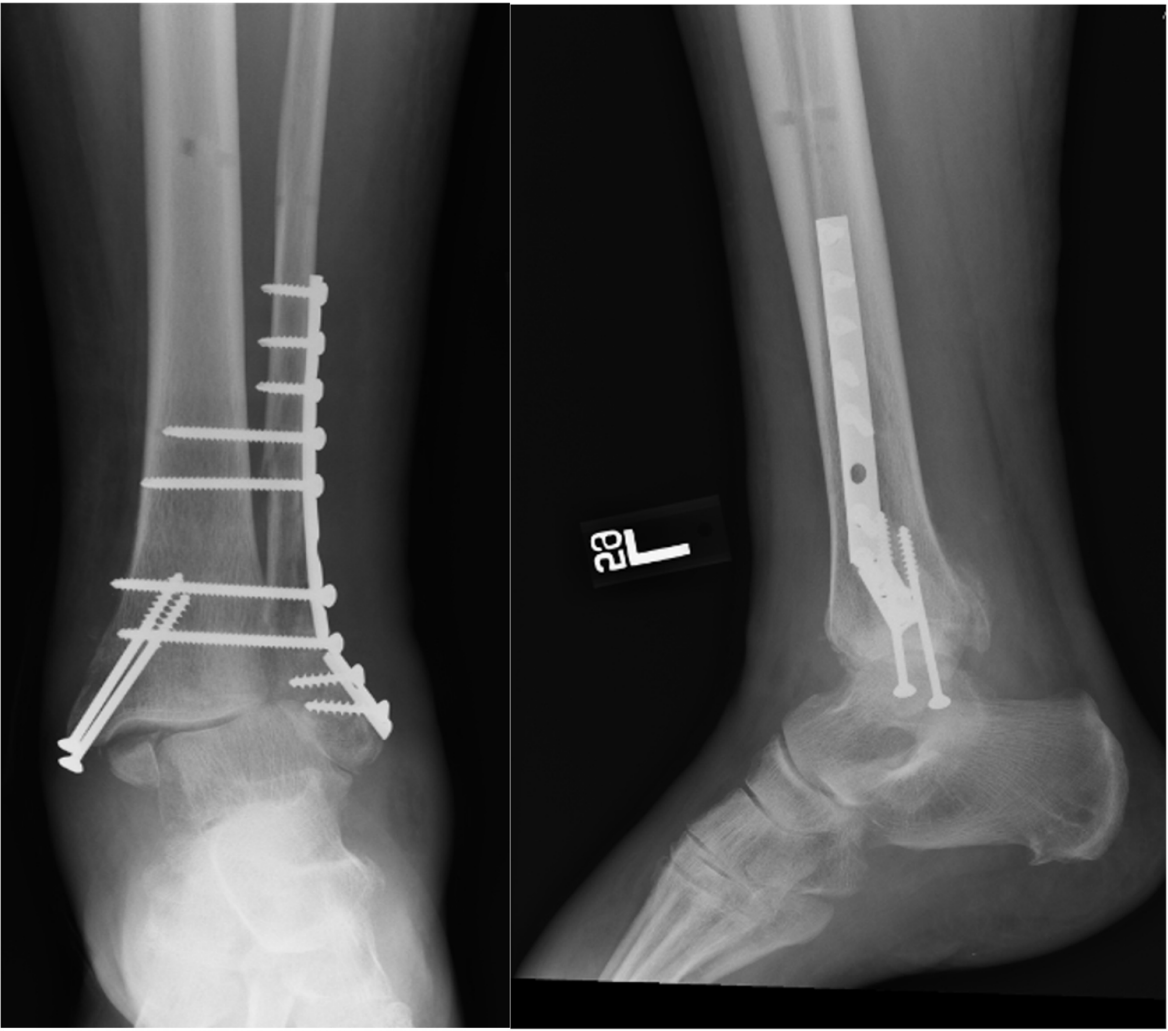
Postoperative radiographs demonstrating open reduction and internal fixation (ORIF) for an unstable ankle fracture. Standard anteroposterior and lateral views show anatomic restoration of the tibiotalar joint with plate-and-screw fixation of the fibula and supplemental fixation of associated medial or posterior malleolar fragments.

**Figure 2.**
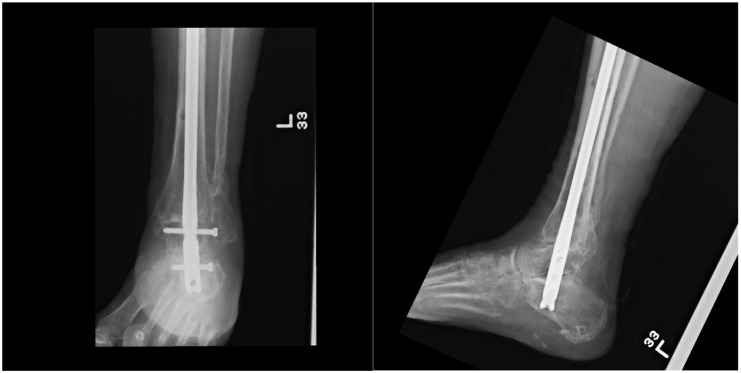
Representative postoperative radiographs showing tibiotalocalcaneal (TTC) fusion with a retrograde intramedullary nail. Anteroposterior and lateral views demonstrate hindfoot alignment and stable nail fixation across the ankle and subtalar joint.

Forty-five total patients met inclusion criteria for the study. The median follow-up time for the ORIF cohort was 419 days (IQR 135–692) and 266 days (IQR 90–349) for the TTC fusion cohort. Twenty-six patients underwent ORIF (Figure 1), and 19 patients underwent TTC fusion (Figure 2). Of the 19 patients in the TTC fusion cohort, fourteen (73.7%) underwent joint preparation prior to TTC nail placement. ORIF was not performed with joint preparation. The sex, race, and ethnicity (if available) of each patient was recorded (Table 1). In the ORIF cohort, 19 (73.1%) patients were female, and 7 (26.9%) patients were male, and in the TTC fusion cohort, 10 (52.6%) patients were female and 9 (47.4%) were male (*P* = .21 between cohorts). Twenty-five (96.2%) total patients identified as White, and 1 (3.8%) patient identified as Black or African American. In the TTC fusion cohort, all 19 (100%) patients identified as White. Additionally, age at the time of surgery, insurance coverage, and BMI of patients within each cohort were recorded (Table 1). There was not a significant difference in age (*P* = .31) and BMI (*P* = .91) between the 2 cohorts.

**Table 1.** Basic Demographics of Patients With Neuropathy of the Lower Extremity and Ankle Fracture Requiring Operative Management With ORIF vs TTC Fusion.

	Fixation Type			
	ORIF (n = 26)	TTC Fusion (n = 19)	Total (N = 45)	*P* Value
Sex, n (%)				.21^a^
Female	19 (73.1)	10 (52.6)	29 (64.4)	
Male	7 (26.9)	9 (47.4)	16 (35.6)	
Race, n (%)				>.99^a^
Black or African American	1 (3.8)	0 (0.0)	1 (2.2)	
White	25 (96.2)	19 (100.0)	44 (97.8)	
Ethnicity, n (%)				
Not Hispanic/Latino/Spanish	24 (100.0)	18 (100.0)	42 (100.0)	
Missing	2	1	3	
Age, y, median (IQR)	64.5 (60.0, 71.0)	66.0 (61.0, 81.0)	66.0 (61.0, 71.0)	.31^b^
BMI, median (IQR)	36.5 (33.1, 41.0)	35.1 (31.2, 47.4)	36.2 (33.1, 41.8)	.91^b^
Insurance, n (%)				.016^a^
Medicaid	3 (11.5)	0 (0.0)	3 (6.7)	
Medicare	13 (50.0)	17 (89.5)	30 (66.7)	
Private	10 (38.5)	2 (10.5)	12 (26.7)	
Smoking status, n (%)				>.99^a^
Current	2 (7.7)	1 (5.3)	3 (6.7)	
Former	9 (34.6)	6 (31.6)	15 (33.3)	
Never	15 (57.7)	12 (63.2)	27 (60.0)	
Fracture type, n (%)				
Unimalleolar	1 (3.8)	3 (15.8)	4 (8.9)	.33^a^
Bimalleolar	11 (42.3)	7 (36.8)	18 (40.0)	.77^a^
Trimalleolar	11 (42.3)	5 (26.3)	16 (35.6)	.35^a^
Distal fibula fracture	1 (3.8)	3 (15.8)	4 (8.9)	.30^a^
Unknown	2 (7.7)	1 (5.3)	3 (6.7)	>.99^a^
Vascular comorbidities, n (%)	25 (96.2)	17 (89.5)	42 (93.3)	.56^a^
Pulmonary comorbidities, n (%)	4 (15.4)	4 (21.1)	8 (17.8)	.70^a^
Diabetes mellitus, n (%)	26 (100.0)	17 (89.5)	43 (95.6)	.17^a^
Rheumatologic comorbidities, n (%)	4 (15.4)	2 (10.5)	6 (13.3)	>.99^a^
Renal comorbidities, n (%)	5 (19.2)	7 (36.8)	12 (26.7)	.31^a^

Abbreviations: BMI, body mass index; ORIF, open reduction internal fixation; TTC, tibiotalocalcaneal.

a Fisher exact *P* value.

b Kruskal-Wallis *P* value.

### Smoking History

Tobacco use in the preoperative period was explored in both patient cohorts with 3 categories described including current, former, or never smoker. Current smokers were those who were still smoking at the time of the initial operation whereas former smokers were those who previously smoked but were not smoking at the time of the initial operation. Tobacco usage compared among the 2 cohorts were not statistically significant (*P* > .99).

### Fracture Type

In the ORIF cohort, 2 patients (7.6%) had the preoperative diagnosis of a unimalleolar fracture, 11 (42.3%) had bimalleolar fractures, 11 (42.3%) had trimalleolar fractures, 1 (3.8%) had a distal fibular fracture, and 2 (7.7%) had unspecified fractures. Of these fractures, 24 (92.3%) were closed fractures and 2 (7.7%) were open fractures. In patients who underwent TTC fusion, 6 (31.6%) had a unimalleolar fracture, 7 (36.8%) had bimalleolar fractures, 5 (26.3%) had trimalleolar fractures, 3 (15.8%) had distal fibular fractures, and 1 (5.3%) had an unspecified fracture. Of these fractures, 17 (89.5%) were closed fractures and 2 (10.5%) were open fractures. Between ORIF and TTC cohorts, there were no significant differences in any fracture type.

### Comorbidities

In the ORIF group, 26 (100%) of patients had diabetes mellitus, 25 (96.2%) had vascular comorbidities, 4 (15.4%) had pulmonary comorbidities, 4 (15.4%) had rheumatologic comorbidities, and 5 (19.2%) had renal comorbidities. In the TTC fusion cohort, 17 (89.7%) of patients had diabetes mellitus, 17 (89.5%) had vascular comorbidities, 4 (21.1%) had pulmonary comorbidities, 2 (10.5%) had rheumatologic comorbidities, and 7 (36.8%) had renal comorbidities. The rates of the comorbidities did not differ between cohorts (Table 1).

### Weightbearing Status

Weightbearing status at 2 weeks included 3 categories: NWB, TTWB, and WBAT. At the 2-week postoperative period, 21 (80.8%) ORIF patients were NWB, 3 (11.5%) were TTWB, and 2 (7.7%) were WBAT. The weightbearing status of TTC fusion patients at 2 weeks included 7 (36.8%) patients who were NWB, 4 (21.1%) that were TTWB, and 8 (42.1%) that were WBAT. There was a statistically significant difference in the 2-week postoperative weightbearing status (*P* = .007) (Table 2). The weightbearing status at 4-6 weeks (*P* = .16) and 12 weeks (*P* = .29) postoperatively was not statistically significant between the 2 cohorts.

**Table 2.** Complication Profile for Patients With Neuropathy of the Lower Extremity and Ankle Fracture Following ORIF or TTC Fusion Surgery.

	Fixation Type			
	ORIF (n = 26)	TTC Fusion (n = 19)	Total (N = 45)	*P* Value
Reoperation, n (%)	14 (53.8)	10 (55.6)	24 (54.5)	>.99^a^
Opioid pain medication, n (%)	21 (80.8)	15 (78.9)	36 (80.0)	>.99^a^
Weightbearing status: 2 wk, n (%)				.007^a^
NWB	21 (80.8)	7 (36.8)	28 (62.2)	
TTWB	3 (11.5)	4 (21.1)	7 (15.6)	
WBAT	2 (7.7)	8 (42.1)	10 (22.2)	
Weightbearing status: 4-6 wk, n (%)				.16^a^
NWB	15 (57.7)	6 (33.3)	21 (47.7)	
TTWB	2 (7.7)	0 (0.0)	2 (4.5)	
WB	8 (30.8)	10 (55.6)	18 (40.9)	
WBAT	1 (3.8)	2 (11.1)	3 (6.8)	
Missing	0	1	1	
Weightbearing status: 12 wk, n (%)				.29^a^
NWB	1 (3.8)	3 (16.7)	4 (9.1)	
WB	25 (96.2)	15 (83.3)	40 (90.9)	
Missing	0	1	1	
Infection, n (%)	7 (26.9)	8 (42.1)	15 (33.3)	.35^a^
Wound dehiscence/poor wound healing, n (%)	11 (42.3)	9 (47.4)	20 (44.4)	.77^a^
Readmission to ED or inpatient after procedure, n (%)	11 (42.3)	7 (36.8)	18 (40.0)	.77^a^
Nonunion, n (%)	3 (11.5)	3 (16.7)	6 (13.6)	.68^a^
Broken or failed hardware, n (%)	11 (42.3)	4 (22.2)	15 (34.1)	.21^a^
Number of operations				.61^b^
Median (IQR)	2.0 (1.0, 3.0)	2.0 (1.0, 5.0)	2.0 (1.0, 4.0)	
Range	1.0, 6.0	1.0, 7.0	1.0, 7.0	

Abbreviations: ED, emergency department; NWB, nonweightbearing; ORIF, open reduction internal fixation; TTC, tibiotalocalcaneal; TTWB; toe-touch weightbearing; WB, weightbearing; WBAT, weightbearing as tolerated.

a Fisher exact *P* value.

b Kruskal-Wallis *P* value.

### Operative Outcomes

Fourteen ORIF patients (53.8%) and 10 TTC fusion patients (55.6%) required reoperation following primary fixation, a difference that was not statistically significant between the 2 cohorts (odds ratio [OR] 0.9, 95% CI 0.28-3.12, *P* > .99) (Table 2). Opioid pain medications were used for most patients, with 21 (80.8%) ORIF patients and 15 (78.9%) TTC fusion patients requiring them. There was not a statistically significant difference in opioid pain medication usage between the 2 cohorts (*P* > .99). There was no significant difference between the ORIF and TTC fusion cohort for infection (OR 0.5, 95% CI 0.14-1.78, *P* = .35), wound dehiscence (*P* = .77), or readmission (*P* = .77). There was no significant difference in rates of nonunion (*P* = .68), or broken or failed hardware (*P* = .21) between the 2 cohorts (Table 2).

Fourteen patients in the ORIF cohort and 10 patients in the TTC fusion cohort underwent reoperations. The median number of reoperations was 2 for both cohorts, with a maximum of 6 in the ORIF cohort and 7 in the TTC fusion cohort (Table 2). Among patients requiring reoperation, there were no significant differences between the 2 groups for any intervention during reoperation, including amputation (7.1% vs 40%, OR 0.1, 95% CI 0.01-1.27, *P* = .12), except for the percentage who received an antibiotic-coated or spacer intramedullary rod (14.3% vs 60%, *P* = .032) (Table 3).

**Table 3.** Outcomes for Patients With Neuropathy of the Lower Extremity and Ankle Following ORIF or TTC Fusion Surgery Who Underwent Reoperations.

	Fixation Type			
	ORIF (n = 14)	TTC Fusion (n = 10)	Total (N = 24)	*P* Value
Removal of hardware, n (%)	11 (78.6)	9 (90.0)	20 (83.3)	.61^a^
Incision and drainage, n (%)	7 (50)	7 (70)	14 (58.3)	.42^a^
Wound vacuum, n (%)	4 (28.6)	1 (10.0)	5 (20.8)	.36^a^
External fixator, n (%)	4 (28.6)	1 (10.0)	5 (20.8)	.36^a^
Amputation, n (%)	1 (7.1)	4 (40.0)	5 (20.8)	.12^a^
Abx-coated spacer IM rod, n (%)	2 (14.3)	6 (60.0)	8 (33.3)	.032^a^
Periprosthetic fracture ORIF, n (%)	0 (0.0)	1 (10.0)	1 (4.2)	.42^a^
Converted to a fusion, n (%)	7 (50.0)	3 (33.3)	10 (43.5)	.67^a^

Abbreviations: Abx, antibiotic; IM, intramedullary; NWB, nonweightbearing; ORIF, open reduction internal fixation; TTC, tibiotalocalcaneal.

^a^Fisher exact *P* value.

Additionally, a sub-analysis was conducted to compare outcomes between TTC fusion patients who underwent joint preparation vs those who did not. Although our cohort size was limited, we did not note differences in demographics between the 2 TTC fusion cohorts (Table 4). Similarly, we did not note any differences in postoperative complications following the initial procedure (Table 5) or among revision surgeries (Table 6) for either cohort.

**Table 4.** Demographic Features of Patients Who Underwent TTC Fusion With and Without Joint Preparation.

	Fixation Type			
	TTC Fusion With Joint Prep (n = 14)	TTC Fusion Without Joint Prep (n = 5)	Total (N = 19)	*P* Value
Sex, n (%)				.30^a^
Female	6 (42.9)	4 (80.0)	10 (52.6)	
Male	8 (57.1)	1 (20.0)	9 (47.4)	
Race, n (%)				
White	14 (100.0)	5 (100.0)	19 (100.0)	
Ethnicity, n (%)				
Not Hispanic, Latino, or Spanish origin	13 (100.0)	5 (100.0)	18 (100.0)	
Missing	1	0	1	
Age, median (IQR)	65.5 (61.0, 76.0)	70.0 (68.0, 84.0)	66.0 (61.0, 81.0)	.31^b^
BMI, median (IQR)	36.6 (33.3, 47.7)	33.9 (31.2, 42.6)	35.1 (31.2, 47.4)	.64^b^
Insurance, n (%)				.47^a^
Medicare	13 (92.9)	4 (80.0)	17 (89.5)	
Private	1 (7.1)	1 (20.0)	2 (10.5)	
Smoking status, n (%)				>.99^a^
Current	1 (7.1)	0 (0.0)	1 (5.3)	
Former	4 (28.6)	2 (40.0)	6 (31.6)	
Never	9 (64.3)	3 (60.0)	12 (63.2)	
Vascular comorbidities, n (%)				.47^a^
No	1 (7.1)	1 (20.0)	2 (10.5)	
Yes	13 (92.9)	4 (80.0)	17 (89.5)	
Pulmonary comorbidities, n (%)				>.99^a^
No	11 (78.6)	4 (80.0)	15 (78.9)	
Yes	3 (21.4)	1 (20.0)	4 (21.1)	
Diabetes mellitus, n (%)				.47^a^
No	1 (7.1)	1 (20.0)	2 (10.5)	
Yes	13 (92.9)	4 (80.0)	17 (89.5)	
Rheumatologic comorbidities, n (%)				>.99^a^
No	12 (85.7)	5 (100.0)	17 (89.5)	
Yes	2 (14.3)	0 (0.0)	2 (10.5)	
Renal comorbidities, n (%)				>.99^a^
No	9 (64.3)	3 (60.0)	12 (63.2)	
Yes	5 (35.7)	2 (40.0)	7 (36.8)	

Abbreviations: BMI, body mass index; TTC, tibiotalocalcaneal.

a Fisher exact *P* value.

b Kruskal-Wallis *P* value.

**Table 5.** Complication Profile for Patients Who Underwent TTC Fusion With Joint Preparation vs Those Without Joint Preparation.

	Fixation Type			
	TTC Fusion With Joint Prep (n = 14)	TTC Fusion Without Joint Prep (n = 5)	Total (N = 19)	*P* Value
Reoperation, n (%)				>.99^a^
No	6 (46.2)	2 (40.0)	8 (44.4)	
Yes	7 (53.8)	3 (60.0)	10 (55.6)	
Missing	1	0	1	
Opioid pain medication, n (%)				>.99^a^
No	3 (21.4)	1 (20.0)	4 (21.1)	
Yes	11 (78.6)	4 (80.0)	15 (78.9)	
Weightbearing status: 2 wk, n (%)				.66^a^
NWB	5 (35.7)	2 (40.0)	7 (36.8)	
TTWB	4 (28.6)	0 (0.0)	4 (21.1)	
WBAT	5 (35.7)	3 (60.0)	8 (42.1)	
Weightbearing status: 4-6 wk, n (%)				.79^a^
NWB	5 (38.5)	1 (20.0)	6 (33.3)	
WB	7 (53.8)	3 (60.0)	10 (55.6)	
WBAT	1 (7.7)	1 (20.0)	2 (11.1)	
Missing	1	0	1	
Weightbearing status: 12 wk, n (%)				>.99^a^
NWB	2 (15.4)	1 (20.0)	3 (16.7)	
WB	11 (84.6)	4 (80.0)	15 (83.3)	
Missing	1	0	1	
Infection, n (%)				>.99^a^
No	8 (57.1)	3 (60.0)	11 (57.9)	
Yes	6 (42.9)	2 (40.0)	8 (42.1)	
Wound dehiscence/poor wound healing, n (%)				.63^a^
No	8 (57.1)	2 (40.0)	10 (52.6)	
Yes	6 (42.9)	3 (60.0)	9 (47.4)	
Readmission to ED or inpatient after procedure, n (%)				>.99^a^
No	9 (64.3)	3 (60.0)	12 (63.2)	
Yes	5 (35.7)	2 (40.0)	7 (36.8)	
Nonunion, n (%)				.17^a^
No	12 (92.3)	3 (60.0)	15 (83.3)	
Yes	1 (7.7)	2 (40.0)	3 (16.7)	
Missing	1	0	1	
Broken or failed hardware, n (%)				>.99^a^
No	10 (76.9)	4 (80.0)	14 (77.8)	
Yes	3 (23.1)	1 (20.0)	4 (22.2)	
Missing	1	0	1	
Number of operations				.43^b^
No	14	5	19	
Median (IQR)	1.5 (1.0, 5.0)	5.0 (1.0, 5.0)	2.0 (1.0, 5.0)	
Range	1.0, 7.0	1.0, 6.0	1.0, 7.0	

Abbreviations: ED, emergency department; NWB, nonweightbearing; TTC, tibiotalocalcaneal; TTWB; toe-touch weightbearing; WB, weightbearing; WBAT, weightbearing as tolerated.

a Fisher exact *P* value.

b Kruskal-Wallis *P* value.

**Table 6.** Outcomes for Patients With Neuropathy of the Lower Extremity and Ankle Following TTC Fusion Surgery With and Without Joint Preparation Who Underwent Reoperations.

	TTC Fusion With Joint Prep (n = 7)	TTC Fusion Without Joint Prep (n = 3)	Total (N = 10)	*P* Value
Removal of hardware, n (%)				>.99^a^
No	1 (14.3)	0 (0.0)	1 (10.0)	
Yes	6 (85.7)	3 (100.0)	9 (90.0)	
Wound vacuum, n (%)				.30^a^
No	7 (100.0)	2 (66.7)	9 (90.0)	
Yes	0 (0.0)	1 (33.3)	1 (10.0)	
External fixator, n (%)				>.99^a^
No	6 (85.7)	3 (100.0)	9 (90.0)	
Yes	1 (14.3)	0 (0.0)	1 (10.0)	
Amputation, n (%)				>.99^a^
No	4 (57.1)	2 (66.7)	6 (60.0)	
Yes	3 (42.9)	1 (33.3)	4 (40.0)	
Abx-coated spacer IM rod, n (%)				>.99^a^
No	3 (42.9)	1 (33.3)	4 (40.0)	
Yes	4 (57.1)	2 (66.7)	6 (60.0)	
Periprosthetic fracture ORIF, n (%)				>.99^a^
No	6 (85.7)	3 (100.0)	9 (90.0)	
Yes	1 (14.3)	0 (0.0)	1 (10.0)	
Converted to a fusion, n (%)				.46^a^
No	3 (50.0)	3 (100.0)	6 (66.7)	
Yes	3 (50.0)	0 (0.0)	3 (33.3)	
Missing	1	0	1	

Abbreviations: Abx, antibiotic; IM, intramedullary; ORIF, open reduction internal fixation; TTC, tibiotalocalcaneal.

a Fisher exact *P* value.

## Discussion

In this retrospective analysis, we found no significant difference in complication rates between ORIF and TTC fusion for neuropathic ankle fractures, although TTC fusion was associated with earlier weightbearing. There is limited orthopaedic literature comparing the clinical outcomes of patients with progressive neuropathic ankle fractures, defined by interval clinical or radiographic progression of deformity or instability in the setting of peripheral neuropathy, undergoing TTC fusion vs ORIF. The purpose of this study was to determine the outcomes and the complication profile following operative fixation of a neuropathic fracture for those undergoing either procedure. Overall, the results of this study with a sample size of 45 patients suggest that although TTC fusion is associated with earlier weightbearing capabilities than patients undergoing ORIF, no clear differences in postoperative outcomes were detected between the 2 cohorts, acknowledging that the study was underpowered to demonstrate equivalence.

Recent literature shows that TTC fusion is a viable and possibly better alternative to traditional ORIF because it offers earlier weightbearing capabilities that may provide better patient outcomes.^16,17^ As expected, our results show that there was a significant difference in weightbearing capabilities between TTC fusion patients at the 2-week postoperative time period, but not at the 4- to 6-week or 12-week time period when compared to the ORIF cohort. However, there was no clear difference in postoperative outcomes such as infection, poor wound healing, ED readmissions, reoperation, or opioid medication prescription between the 2 cohorts. As such, postoperative weightbearing differences between the 2 procedures may not play an influential role in patient healing and recovery as previously suggested.^18^ Instead, these results may suggest that orthopaedic surgeons trust TTC fusion constructs more than ORIF constructs and allow TTC fusion patients to weightbear earlier in the recovery process, although these differences in weightbearing timeline may not impact clinical outcomes. Therefore, the utility and optimal time frame for which patients can start to weightbear after ankle arthrodesis must be further elucidated.

Although limited data exist comparing the 2 procedure types, ORIF may be associated with significantly increased rates of postoperative complications when compared to TTC fusion.^19^ Specifically, ORIF for neuropathic ankle fractures is associated with high rates of complications especially in elderly patients with diabetes mellitus, peripheral neuropathy, peripheral vascular disease, smoking, obesity, and/or osteoporosis for which TTC fusion may be more suitable.^16^ However, our results showed that there was not a significant difference in the patient’s ages, smoking history, or comorbidities (Table 1) between the cohorts, and more importantly, there was not a significant difference in outcomes. Operative intervention via TTC fusion vs ORIF, if indicated, may also be dependent on mechanism and open vs closed nature of the injury, presence or absence of fracture blisters/ulcerations, quality of the soft tissues, and location of the planned incision. Our findings align with previous data that ORIF in diabetic patients is a reasonable option for neuropathic fractures and that TTC fusion may not always be the best option for these patients.^20^

There is currently a knowledge gap in which procedure type, either ORIF or TTC fusion, provides better outcomes specifically for diabetic patients.^21^ Given the strength and functionality of intramedullary nails, TTC fusions are often performed in patients with many comorbidities who have complex fractures, especially those with Charcot neuroarthropathy. This finding may seemingly skew the risk of postoperative complications and revisions in TTC fusions.^15^ Interestingly, all 19 of the patients in the TTC fusion group and 92.3% of patients in the ORIF group had a diabetes diagnosis (Table 1), although the degree of glycemic control in these patients remains unknown. The rate of complications such as infection, poor wound healing, ED readmission, and reoperation in either group was relatively high (Table 2), a finding that should be expected in diabetic patients undergoing any surgical procedure because of the risk of poor wound healing and infection. Therefore, the high reoperation rates observed in both cohorts highlight the inherent complexity of neuropathic ankle fractures, regardless of fixation strategy. Although TTC fusion and ORIF showed no clear difference in neuropathic fractures,^22,23^ the risk of postoperative complications remains high regardless of procedure type and must be considered before performing ankle arthrodesis in diabetic patients.

The present study had some limitations. First, the sample size was relatively small (n = 45), which may increase the risk of type II error and random variations. Given the retrospective design of the study, we could not conclude the severity of the patient’s ankle injury or the degree of peripheral neuropathy before surgery, nor could we determine the rationale for electing for ORIF vs TTC fusion. TriNetX may not comprehensively capture all relevant outcomes such as detailed fracture classifications. As the platform integrates data from diverse electronic medical record systems, variability in coding practices and documentation across institutions may introduce inconsistencies in diagnoses and procedural data. Additionally, there was not a controlled weightbearing protocol among the cohorts, so it is unclear what mobility was afforded to patients in either cohort or how well the patients adhered to their weightbearing status in the postoperative period. Likewise, there was limited information available as to how a patient’s weightbearing status was determined following surgery. Although there were no significant differences in comorbidities between the 2 cohorts, patients were not propensity matched and therefore the patient outcomes may be a result of factors other than the type of surgery the patient received. Future studies should examine temporal trends in TTC fusion outcomes, include larger and more diverse patient populations, and assess functional outcomes following ORIF or TTC fusion.

## Conclusion

Our data suggest there is no clear difference detected in postoperative outcomes for patients with neuropathic fractures who underwent TTC fusion or ORIF, in this cohort, independent of weightbearing status throughout recovery. In patients who may benefit from early mobilization for reasons unrelated to their fracture, TTC nailing represents a viable option that can facilitate earlier functional mobility. However, if a patient is deemed not ideal for TTC fusion due to weightbearing or operative concerns, then ORIF may provide similar outcomes for patients with neuropathic fractures.

## Supplemental Material

sj-pdf-1-fao-10.1177_24730114261425629 – Supplemental material for Retrospective Comparison of Complication Rates Following Tibiotalocalcaneal (TTC) Nail Fusion vs ORIF for Neuropathic Ankle Fractures Supplemental material, sj-pdf-1-fao-10.1177_24730114261425629 for Retrospective Comparison of Complication Rates Following Tibiotalocalcaneal (TTC) Nail Fusion vs ORIF for Neuropathic Ankle Fractures by Spencer C. DeMedal, Kelly Dopke, Kelan Queenan, Samantha N. Olson, Michael F. Levidy, Tonya S. King, Jana Davis and Michael C. Aynardi in Foot & Ankle Orthopaedics
